# Opposing effects on glutathione and reactive oxygen metabolites of sex, habitat, and spring date, but no effect of increased breeding density in great tits (*Parus major*)

**DOI:** 10.1002/ece3.663

**Published:** 2013-07-11

**Authors:** Caroline Isaksson

**Affiliations:** Edward Grey InstituteDepartment of Zoology, University of OxfordOxford, UK

**Keywords:** Antioxidant, ecophysiology, hydroperoxides, life history, oxidative stress, passerine

## Abstract

Oxidative stress (i.e., more oxidants than antioxidants) has been proposed as a proximate currency in life-history trade-offs, which if studied in an ecological setting allow a more realistic perspective on the origin and evolution of trade-offs. Therefore, the aim here was to investigate the impact of ecological and individual factors for variation in markers of oxidative stress using both experimental and correlational data. Total glutathione (tGSH), oxidized glutathione (GSSG), plasma antioxidant capacity (OXY), and plasma-reactive oxygen metabolites (ROM) were measured in more than 700 breeding great tits (*Parus major*). The main results revealed a pronounced sex difference, with females having lower ROM and OXY, but higher tGSH compared with males. In addition, birds breeding in the evergreen areas had higher tGSH compared with those in the deciduous habitat, but the experimentally manipulated breeding density had no significant effect on any of the redox markers. Independent of the sex differences, the larger the reproductive investment the lower the ROM of both males and females. Taken together, the extracellular markers – ROM and OXY – revealed similar results and were highly correlated. Interestingly, the direction of their effects was in the opposite direction to the endogenously synthesized tGSH and GSSG. This highlights the need to combine extracellular markers with endogenously synthesized antioxidants to understand its implications for the origin and evolution of trade-offs in an ecological setting.

Oxidative stress has been proposed as a proximate currency in life-history trade-offs, which if studied in an ecological setting allow a more realistic perspective on the origin and evolution of trade-offs. Here multiple markers of oxidative stress were analysed in wild great tits. The results reveal that the endogenously synthesized antioxidant glutathione and markers of plasma oxidative stress are affected in opposing directions with regard to sex, habitat type, and spring date. Clutch size was negatively associated with oxidative damage, which suggests that those with high reproductive investment can combat physiological costs linked to oxidative stress. The experimentally manipulated breeding density did not influence oxidative stress physiology. The study highlights the need to measure multiple markers to understand the role of oxidative stress in limiting the expression of life-history traits and trajectories in different ecological contexts.

## Introduction

There is increasing interest among ecologists in the physiological processes that underlie individual differences in traits that influence fitness. This is because disentangling the mechanisms that mediate life histories may allow a more realistic perspective on the origin and evolution of trade-offs, the ecological conditions that mediate those trade-offs, and thus how individual variation in life-history trajectories is maintained (e.g., Lessells 27; Monaghan et al. 30; Isaksson et al. 23,b).

In recent years, the mechanism that has received the most interest in this context is arguably oxidative stress, that is, a surplus of the reactive oxidants (such as reactive oxygen species [ROS]) to the protective antioxidants (such as glutathione and α-tocopherol). The focus on oxidative stress arises from the paradox that oxygen and ROS are crucial for cellular life, but that ROS are at the same time detrimental to lipids, proteins, and DNA unless they are detoxified. For example, ROS are actively produced and released during the initial phases of infection (Sorci and Faivre 41), but they are also produced as a by-product during aerobic respiration (Cadenas and Davies 10). In addition, ROS are also used as signal molecules between cells, crucial for triggering, for example, female reproduction (Agarwal et al. 1). It has been shown that experimentally increased reproduction increases the level of oxidative damage and decreases resistance to an oxidative attack (Alonso-Alvarez et al. 3; Wiersma et al. 50; Christe et al. 11), suggesting that a metabolically demanding trait like reproduction may indeed increase ROS leakage (but see Selman et al. 39). Furthermore, oxidative stress is also predicted to accumulate and/or increase with age (Finkel and Holbrook 16), providing a physiological memory and a link between current and future performance (reviewed in Monaghan et al. 30; Isaksson et al. 23,b). However, studies investigating the relationship between longevity and oxidative stress are not conclusive and more research is needed in this area (e.g., Robert and Bronikowski 38; Speakman and Selman 42).

In addition, environmental factors can directly or indirectly influence the oxidative stress status of wild animals (Isaksson et al. 21; Isaksson and Andersson 20; Nussey et al. 32; Costantini 12; van de Crommenacker et al. 13, 14). Diet quality and quantity such as intake of dietary antioxidants or essential building blocks for endogenously synthesized antioxidants, along with intake of toxins that are active as oxidants are examples of how environmental differences can influence the oxidative stress status directly (see Isaksson et al. 23a). Other environmental factors may be proxies for linked differences in, for example, diet, behaviour, reproduction, or infections (e.g., Christe et al. 11; Isaksson et al. 25).

The lack of comprehensive studies of variation in extracellular and endogenously synthesized antioxidants and oxidants in ecological contexts leaves us poorly equipped to interpret the functional significance of laboratory studies, where phenotypic and environmental variation typically is experimentally kept to a minimum. The lack of data on natural variation is also a problem with respect to the use of measures of oxidative stress as biomarkers in the wild (Hõrak and Cohen 19).

To address these issues, a large study of individual variation in the endogenously synthesized antioxidant system – glutathione, and extracellular measures of oxidative stress was conducted using a wild population of great tits (*Parus major*). The population is particularly useful for testing how environmental variation affects oxidative stress as nest boxes had been set up to create a factorial design with two different habitat types (deciduous- vs. evergreen-dominated habitat) crossed with two different breeding densities. The two habitat types correspond to a large difference in caterpillar abundance (i.e., deciduous habitat has higher abundance compared with the evergreen area, see Data S1, Figs. S1 and S2). The factor of habitat type is likely to affect food intake, investment in reproduction, and time spent foraging, all of which are known to influence oxidative stress physiology (e.g., Isaksson and Andersson 20; Slos et al. 40; Costantini 12; van de Crommenacker et al. 13). The experimentally manipulated breeding density is likely to increase intraspecific competition and interactions, and many life-history traits in great tits show density dependence (e.g., Wilkin et al. 51). High breeding density in this population has also recently been shown to increase avian malaria prevalence (Isaksson et al. 25), which has been shown to increase plasma-reactive oxygen metabolites (ROM; van de Crommenacker et al. 14; see also Bertrand et al. 6). Thus, increased breeding density is predicted to increase oxidative stress. In addition to habitat type and breeding density, spring date (within year variation in timing of breeding), clutch size, sex, age, and body condition were investigated.

## Materials and Methods

### Study site and habitat differences

This study was carried out during April–June in 2009 and 2010 in a nest-box population of great tits (*P. major*). The study site, Bagley woods, is a 250-ha woodland (51°42′N, 5°37′W), with a total of 513 nest boxes distributed across a mixed matrix of plantations of either evergreen trees (mainly pine *Pinus* and larch *Larix*,* n*_plots_ = 4) or deciduous trees (mainly oak *Quercus* dominated*, n*_plots_ = 8). During the breeding season, great tits are dependent on Lepidoptera caterpillars as a food source for raising their chicks, and caterpillar abundance is directly linked to oak abundance (Perrins 33). To confirm this habitat difference in insect abundance between the two habitat types, 26 water basins (70 × 45 cm) were used as insect traps, emptied, and caterpillars were counted (*n *= 208, each trap was emptied eight times) over the course of the whole breeding season (see Data S1, Figs. S1 and S2). Briefly, the results confirmed previous studies that habitats with high abundance of deciduous trees have a high caterpillar abundance compared with evergreen habitats (*n *= 208, df_den_ = 27.23, *F* = 27.23, *P *< 0.0001). Furthermore, the nest boxes were experimentally placed at two densities (high density = HD, *n*_plots_ = 6, and low density = LD, *n*_plots_ = 6) in similar sized areas (approximately 68 and 18 boxes in HD and LD plots, respectively) and cross-factored with the two habitat types. The density arrangements produce average densities of 23.3 and 12.3 great tit nests per HD and LD plot, respectively.

### Study species and sampling

During breeding, adult great tits (*n *= 769) were captured with nest-box traps while feeding their young (between 6 and 10 days after hatching). However, sample sizes are reduced because blood samples were not obtained from all individuals, thus the sample size are reduced to 743 for tGSH and GSSG (2009: *n*_females_ = 167, *n*_males_ = 141; 2010: *n*_females_ = 229, *n*_males_ = 206) and 706 for ROM and antioxidant capacity (OXY) (2009: *n*_females_ = 149, *n*_males_ = 127; 2010: *n*_females_ = 221, *n*_males_ = 209). Furthermore, 112 adult birds were caught and sampled in both years; to control for variation generated by bird identity, this was included as a random factor (see below). Tarsus length was measured with a digital caliper (±1 mm) and body mass with a digital balancer (±0.01 g). Age (1 year and older) was determined by plumage characteristics (Svensson 44). A heparinized syringe was used to draw approximately 110 μL blood from the jugular vein.

### Oxidative stress assays

The antioxidant capacity was analyzed by measuring plasma antioxidant potential (OXY-Adsorbent kit, Diacron international, Grossetto, Italy) and total glutathione (tGSH) from blood cells. As an indication of oxidant status, plasma-reactive oxygen metabolites (d-ROM kit, Diacron international) and cellular oxidized glutathione (GSSG) were measured. For the oxidative stress assays, two different approaches were used in the field: (1) for glutathione (GSH) assays, 40 μL was diluted with 2 μL EDTA and then immediately frozen in liquid nitrogen in the field. (2) The rest (approximately 60 μL) was kept cool on top of ice until it was centrifuged (at 1800 rpm for 10 min) 2–3 h later, and then the plasma was frozen in liquid nitrogen. Samples were stored in a −80°C freezer until assayed (~2–3 months later).

#### tGSH and GSSG

Glutathione is the most abundant intracellular antioxidant, and probably the most important (Halliwell and Gutteridge 18). The reduced GSH detoxifies hydrogen peroxide and lipid hydroperoxides which are major cellular ROS and generates GSSG along with water and alcohol. The GSSG/tGSH is often used as an indicator of oxidative stress. The protocol was slightly modified from Baker et al. (5) and Vandeputte et al. (47). Briefly, whole blood was diluted with 5% 5-sulfoasalicylic acid (SSA) and then centrifuged. The samples were diluted with GSH buffer (143 mmol/L NaH_2_PO_4_ and 6.3 mmol/L EDTA, pH 7.4) and divided into two tubes. In one tube, 4-vinylpyridine (4-vp) was added and incubated for 1 h at 37°C. A plate (one row at the time) was prepared with sample and reaction mix (GSH buffer, 5.5′-dithio-bis(2-nitrobenzoic acid) [DTNB] and NADPH), and glutathione reductase. All samples were run in duplicates and the color generated was immediately measured on a Infinite M200 plate reader (Tecan Group Ltd., M\xE4nnedorf, Switzerland) plate reader at 412 nm for 5 min. The obtained changes (kinetic mode) were compared with daily made GSH and GSSG standards of known concentrations. All chemicals used were purchased from Sigma-Aldrich (Gillingham, Dorset) and Melford (Ipswich; Suffolk), U.K. The assays were further validated by measuring a subset of individuals twice. The across- and within-assay repeatability was tested by using a restricted maximum likelihood mixed model with individual identity as a random factor (Nakagawa and Schielzeth 31). The across-assay repeatability for tGSH was 78% (tGSH, *n* = 54, variance components ± SE, ID: 0.29 × 10^−3^ ± 0.09 ×10^−3^,residual: 0.08 × 10^−3^ ± 0.02 × 10^−3^), and for GSSG it was 71% (GSSG, *n* = 48, variance components ± SE, ID: 10.60 × 10^−6^ ± 3.79 × 10^−6^, residual: 4.22 × 10^−6^ ± 1.23 × 10^−6^). Within-assay repeatability was 98% and 95% for tGSH and GSSG, respectively.

#### OXY-adsorbent and d-ROM kit

Antioxidant capacity test assesses the antioxidant power of plasma by measuring the ability to resist a massive oxidant attack. The plasma barrier includes exogenous (e.g., tocopherols, carotenoids, and flavonoids) and endogenous (e.g., proteins, bilirubin, uric acid, cholesterol, and GSH) compounds. A low value indicates a low antioxidant barrier. ROMs are an indirect measure of hydroperoxides (ROOH) in the sample. Hydroperoxides are both active oxidants (although more stable than other ROS) and generated by the oxidation of several biological substrates, thus a marker of oxidants as well as oxidative damage (e.g., Alberti et al. 2). However, there are some concerns that ROM assays are affected by ceruloplasmin and are insensitive to changes (e.g., Lindschinger et al. 28; Erel 15). Both kits were purchased from Diacron (Grosseto, Italy). Briefly, for OXY plasma samples were diluted and then an oxidant solution (HClO) was added. The mix was then incubated and a chromogen was added. For the ROM assay, a master mix with the active substance was prepared and mixed with a small plasma sample. The mixture was then incubated. Both ROM and OXY samples were analyzed at 490 nm (Biotech Power, Northwest Science Ltd., Billings, MT). Each plate contained a blank and calibrator. The final concentration of the OXY test was calculated according to the formula provided by the manufacturer and expressed as μmol HClO/mL plasma. The repeatability across assays was 53% (OXY, *n *= 56, variance components: ID = 0.53, residual = 0.47). Within-assay repeatability was 97%. The final d-ROM calculations were expressed as mg H_2_O_2_/dL. For dROM assay, the across-assay repeatability was 93% (dROM, *n *= 64, variance components ± SE: ID = 3.79 ± 0.29, residual = 0.28 ± 0.05) and within assay repeatability was 99%.

### Statistical analyses

Each year the samples were analyzed over a 1.5-month period. Although degradation of biomolecules should be of minor issue when frozen in −80°C (as done here), there was a significant negative association for tGSH (tGSH from 2009: *r *= −0.37 *P *< 0.0001), GSSG (GSSG from 2009: *r *= −0.22, *P *< 0.0001), and OXY (OXY from 2009: *r* = −0.17, *P *= 0.002) with laboratory date. ROM was unaffected (ROM from 2009: *r *= −0.04, *P *= 0.447). Similar results were revealed for the 2010 samples. Thus, to control for this effect of time, tGSH, GSSG, and OXY were standardized to a mean of zero and a standard deviation of one for each laboratory day (Quinn and Keough 35). The mean number of samples analyzed per day was 31.6 ± 3.3 for tGSH and GSSG and 51.6 ± 6.3 for ROM and OXY.

Four generalized linear mixed models (GLMM) were performed with the physiological markers as response variables using JMP 9 (SAS Institute. Inc., Cary, NC). Backward elimination of parameters with a *P* > 0.10 was used to reduce the models, starting with the interactions and then with the least significant variables. As fixed effects, habitat type, nest-box density, sex, and age (1 year and older) were included. Clutch size and body condition (residuals from body weight–tarsus regression) were included as covariates. Sampling date was mean centered by year and both the linear and quadratic effects of spring date were included. Spring date indicates the date of blood sampling which is standardized for timing of breeding, that is, the earlier the blood sample was taken the earlier timing of breeding relative to the rest in the population. The biological relevant interactions: sex × clutch size and habitat type × clutch size were included. Bird identity, habitat plot (12 plots), and nest box nested within habitat plot were fitted as random effects. Those random effects that gave negative variance components, that is, explained zero variation were removed from the final models. In addition, regression analysis was conducted to investigate covariance between social partners.

## Results

### ROM and OXY

**Figure 1:**
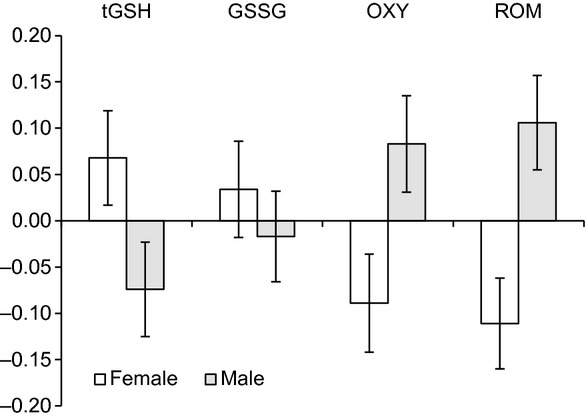
Total glutathione (tGSH), oxidized glutathione (GSSG), plasma antioxidant capacity (OXY), and reactive oxidative metabolites (ROM) of male and female great tits. All values are standardized for laboratory day (see Material and Methods section) and presented as means ± standard error.

**Figure 2:**
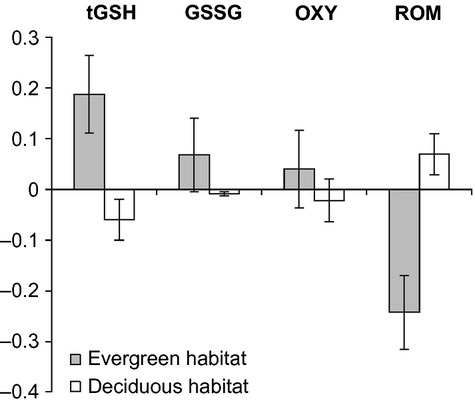
Total glutathione (tGSH), oxidized glutathione (GSSG), plasma antioxidant capacity (OXY), and reactive oxidative metabolites (ROM) of birds breeding in deciduous (abundance of caterpillars) and evergreen habitats (caterpillars are scarce). All values are standardized for laboratory day (see Material and Methods section) and presented as means ± standard error.

Sex, clutch size, date, and (date)^2^ were all strong predictors of ROM (see Table 1). Females had significantly lower concentration of ROM than males (LSM ± SE: females = 2.209 ± 0.134; males = 2.509 ± 0.136; Fig. 1). Great tits with larger reproductive investment, that is, clutch size had a lower ROM (*P *= 0.002, see Table 1, males: *n *= 336, *r* = −0.122, *P *= 0.021, females: *n *= 370, *r *= −0.091, *P *= 0.070). Females laid significantly larger clutches when breeding in the deciduous areas compared with the evergreen areas analyses of variance (ANOVA: *F*_1,704_ = 7.045, *P* = 0.008; mean ± SE: deciduous habitat: 8.69 ± 0.08; evergreen habitat: 8.18 ± 0.15 eggs). However, the number of fledglings raised did not differ (ANOVA: *F*_1,704_ = 0.564, *P *= 0.453; mean ± SE: deciduous habitat: 6.50 ± 0.13; evergreen habitat: 6.69 ± 0.18 chicks). If clutch size was replaced with brood size in the GLMM, the same results were obtained for ROM. ROM was also positively associated with date and negatively with date squared. Nest box nested within habitat plot explained 10.74% of the variation and plot 5.91%. A post hoc Student's *t*-test of the plot effect revealed a difference specifically between one evergreen area and two deciduous areas. Thus, the variation induced by habitat is included in the random effect of plot rather than the fixed factor of habitat type (see Fig. 2 and Table 1). Furthermore, the nest-box effect was supported by a strong positive covariance in both ROM and OXY between the great tit partners (ROM: *n *= 315, *r *= 0.293, *F*_1,313_ = 29.394, *P *< 0.0001; OXY: *n *= 321, *r *= 0.186, *F*_1,319_ = 11.383, *P *= 0.0008).

**Table 1:** Statistical summary table for ROM and OXY

	Estimate ± SE	df_den_	*F*-value	*P*-value
ROM (*n *= 706)				
Date	0.04 ± 0.01	670.72	25.04	<0.0001
(date)^2^	−0.01 ± 0.00	548.76	8.35	0.004
Sex (f)	−0.15 ± 0.05	455.43	9.21	0.003
Habitat quality (low)	−0.22 ± 0.12	12.87	3.19	0.098
Clutch size	−0.09 ± 0.03	510.02	9.93	0.002
	Variance comp. ± SE			Variance explained (%)				
*Nest box [Plot](r)*	0.22 ± 0.09			10.74				
*Plot(r)*	0.12 ± 0.07			5.91				
OXY (*n *= 706)				
Date	0.02 ± 0.01	647.69	11.23	0.001
(date)^2^	−0.01 ± 0.00	543.55	6.63	0.010
Sex (f)	−0.09 ± 0.04	472.89	5.79	0.017
	Variance comp. ± SE			Variance explained (%)				
*Nest box [Plot](r)*	0.07 ± 0.04			7.76				
*Plot(r)*	0			0				

A summary table of the final GLMM for reactive oxygen metabolites (ROM) and total antioxidant capacity (OXY) measured in great tit plasma. Among the single parameters, breeding density, age, and body condition had a *P *> 0.1, thus eliminated from the models. For OXY, clutch size and habitat quality were also removed (see more statistical details in Material and Methods section).

The simplest model for OXY included sex, date, and (date)^2^ (Table 1) and revealed the same directions of the effects as ROM. Females had lower OXY than males (LSM ± SE: females = −0.089 ± 0.052; males = 0.082 ± 0.054; Fig. 1), and linear and quadratic function of spring date revealed positive and negative covariance with OXY, respectively. Nest box nested within habitat plot explained 7.76% of the total variation in OXY (see also above).

**Figure 3:**
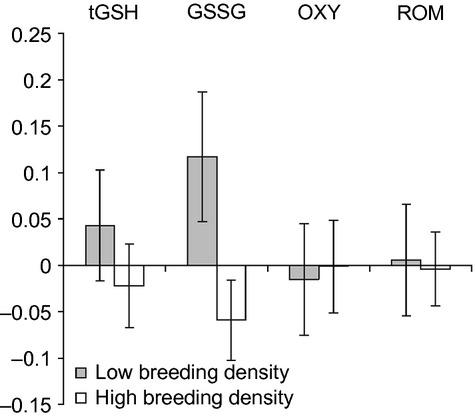
Total glutathione (tGSH), oxidized glutathione (GSSG), plasma antioxidant capacity (OXY), and reactive oxidative metabolites (ROM) of birds breeding in experimentally manipulated low and high densities. All values are standardized for laboratory day (see Material and Methods section) and presented as means ± standard error.

The experimentally manipulated breeding density was removed from the final models of both ROM and OXY (all *P* > 0.1, see Fig. 3).

### tGSH and GSSG in blood

For tGSH, habitat type and sex were significant (Table 2). Birds breeding in the evergreen habitats of low food abundance had higher tGSH than those breeding in the deciduous habitat (LSM ± SE; evergreen = 0.187 ± 0.076, and deciduous habitat = −0.062 ± 0.044, Fig. 2). Furthermore, tGSH was higher in females than in males (Fig. 1). Nest box nested within habitat plot explained 7.31% of the variance in tGSH. However, there was still a positive and highly significant covariance between the male and female partner's in tGSH and GSSG (*n *= 340, tGSH: *F *= 22.163, *P *< 0.0001; and GSSG: *F *= 5.955, *P* = 0.015).

**Table 2:** Statistical summary table for tGSH and GSSG

	Estimate ± SE	df_den_	*F*-value	*P*-value
tGSH (*n* = 743)				
Habitat quality	0.12 ± 0.04	298.29	8.00	0.005
Sex (f)	0.071 ± 0.03	491.59	4.07	0.004
	Variance comp. ± SE			Variance explained (%)				
*Nest box [Plot](r)*	0.07 ± 0.04			7.31				
*Plot(r)*	0			0				
GSSG (*n *= 743)				
Date	0.02 ± 0.01	647.69	11.23	0.001
	Variance comp. ± SE			Variance explained (%)				
*Nest box [Plot](r)*	0.10 ± 0.04			10.49				
*Plot(r)*	0			0				

A summary table of the final GLMM for total (tGSH) and oxidized glutathione (GSSG) measured in great tit whole blood. Among the single parameters, breeding density, age, clutch size, date, date^2^, and body condition had a *P *> 0.1, thus eliminated from the tGSH model. For GSSG, all except spring date were removed (see more statistical details in Material and Methods section).

For GSSG, the single best predictor of this variation was spring date, with birds breeding early in the season having higher GSSG (Table 2). This effect remained the same when tGSH was included as a covariate in the analysis. Nest box nested within habitat plot explained 10.49% of the variation in GSSG.

The experimentally manipulated breeding density was removed from the final models of both tGSH and GSSG (all *P *> 0.1). Birds breeding in high-density areas started to breed later than those breeding in lower densities (*n*_nests_ = 399, ANOVA: *F*_1,397_ = 10.880, *P* = 0.001, LSM ± SE, low breeding density: −1.24 ± 0.30, high breeding density 0.31 ± 0.27), thus the effect of spring date on GSSG confounds the effect of breeding density (see Fig. 3).

## Discussion

The main results in this study reveal that sex, habitat type, and spring date are factors that explain most natural variation in tGSH, GSSG, OXY, and ROM. The experimentally manipulated breeding density did not show any significant effect.

First, adult great tits that are breeding in deciduous-dominated habitats (i.e., higher abundance of caterpillars) have higher levels of ROM compared with birds that breed in evergreen habitats. However, the random effect of habitat plot explained the variation better than the fixed factor of habitat type, thus habitat type did not reach significance in the model (Fig. 2). Perhaps, the most likely explanation of the habitat effect is the difference in food availability between the environments and the plots (see Figs. S1 and S2). The OXY and ROM assays capture several dietary macromolecules such as OXY measures dietary antioxidants (e.g., ascorbic acid and α-tocopherol) and ROM peroxidized dietary amino acids and lipids. Thus, both assays are likely to be influenced by food quantity and/or quality, thereby influencing the circulating levels of ROM and OXY (e.g., Costantini 12; van de Crommenacker et al. 13; see also Costantini 101). However, neither habitat type nor the plot explained any significant variation in OXY. Perhaps the lack of an effect on OXY is due to the low across-assay repeatability compared with ROM. Alternatively, it is less sensitive to environmental changes, because the endogenously synthesized antioxidants can counterbalance the dietary ones (Vertuani et al. 48; see also van de Crommenacker et al. 13). Regarding behavior, the difference in habitat type may influence birds’ individual decision in how much to invest in reproduction, with an increased investment when the opportunity arises via high food availability. This may push individuals over their optimal clutch size, thus an increased physiological challenge despite the higher caterpillar abundance needed to raise a larger brood in the deciduous habitat. Given that the females in the deciduous area lay a larger clutch, but then fail to raise the whole brood, this may be a likely scenario. In contrast to the present results, the direction of the habitat effect on ROM and OXY differs from a recent study on Seychelles warbler *Acrocephalus sechellensis*. In that study, birds that were holding a high-quality territory (i.e., high insect abundance corrected for territory size) had significantly lower absolute and relative ROM (van de Crommenacker et al. 13). The authors interpreted their results as a consequence of higher oxygen metabolism (i.e., higher release of ROS) in low-quality territories due to higher foraging activities to find insects to feed their chicks with. However, the direct link between ROS production and oxidative metabolism has been questioned (Speakman and Selman 42). Possibly, the difference in results is a consequence of difference in habitat structure, breeding opportunities, and family structures (i.e., Seychelles warblers have helpers) between great tits and Seychelles warblers which makes the habitat effect context specific (see Komdeur et al. 26; van de Crommenacker et al. 13, 14).

Furthermore, habitat type also influenced the endogenously synthesized antioxidant – glutathione. However, the effect was in the opposite direction to ROM, with birds in the deciduous habitats having lower tGSH compared with birds in the evergreen habitat, suggesting that birds in the evergreen habitat may have an increased demand of cellular activity and protection, whereas birds in the deciduous habitat can keep cellular homeostasis with a low tGSH. Alternatively, the biosynthesis of glutathione is rate limited by the dietary precursor cysteine, but whether cysteine is limited for birds in the wild and in a deciduous habitat is unknown and requires further investigations (Isaksson et al. 23,b).

In contrast to habitat quality, the experimentally manipulated breeding density did not significantly influence any of the physiological variables. Previously we have shown that high bird densities result in higher prevalence (i.e., presence/absence) of *Plasmodium circumflexum* (a type of avian malaria) which was related to lower oxidation of GSH (Isaksson et al. 25). Thus, it was surprising to not find an effect of breeding density on the GSSG. However, as birds in the high-density areas started to breed later than birds in low-density areas, it is possible that the effect of spring date on GSSG overrides the effect of breeding density (see Fig. 3). Alternatively, yearly spatial and/or individual variation in malaria prevalence overrides the effect of breeding density on GSSG.

Linear and quadratic function of spring date was overall of high importance for explaining variation in all physiological variables except tGSH. Great tits breeding later in the season had higher ROM and OXY, but lower GSSG. Overall, the linear function was a better fit than the quadratic function of spring date. Possible variables that are linked and could contribute to the high importance of spring date or/and timing of breeding (i.e., date for sampling was standardized for hatching of offspring) are temperature, food abundance (i.e., low food abundance early and late in spring, see Data S1), and individual quality (i.e., high-quality individuals breed early, Perrins and McCleery 34).

Furthermore, sex was found to be an overall important explanatory factor. Females had significantly lower extracellular oxidative metabolites and antioxidant defenses, but higher tGSH compared with males. Previously, it has been shown that spotted snow skink females (*Niveoscincus ocellatus*) also have lower levels of ROM compared with males, but no sex difference in OXY was detected (Isaksson et al. 23,b). The sex difference in redox physiology during the breeding season is likely to be mediated via sex steroids, with testosterone increasing and estrogens reducing ROS production (Gupta and Thapliyal 17; Viña et al. 49; Tobler and Sandell 46). Regarding glutathione, it is the most important intracellular antioxidant and the higher levels in females may reflect a higher investment in cellular maintenance or alternatively an upregulation in response to higher cellular ROS during chick feeding (see also Alonso-Alvarez et al. 3; Wiersma et al. 50; Christe et al. 2011). However, the lower ROM levels in females may suggest that they are better protected against oxidative stress than males (e.g., Gupta and Thapliyal 17). Overall, regardless of the mechanism, the finding is likely to reflect a sex difference in behavior, food intake, and/or reproductive physiology that influences antioxidant and oxygen metabolite levels in great tits.

Total GSH is predicted to decline and GSSG and ROM are predicted to increase with age due to cellular senescence (Rebrin and Sohal 37), but here age had no significant effect on physiology. Possibly, this is due to the cross-sectional approach, but more likely is due to the crude pooling of all the individuals that are older than 2 years. In a study of captive partridges (*Alectoris rufa*) of known ages (i.e., 1–8 years old), old partridges had higher GSSG, total OXY, and lipid peroxidation compared with middle-aged partridges (Alonso-Álvarez et al. 4). Unfortunately, these types of analyses were not possible in this data set.

The last parameter tested was clutch size, which was significantly negatively associated with ROM (independent of sex). Similarly, a negative association between ROM and litter size was revealed in female mice that had just given birth; however, after reproduction (during weaning) the association was instead positive (Stier et al. 43). This could indicate that those individuals that have a high reproductive output are the individuals that can initially avoid generation of ROM (high-quality individuals) despite the higher investment, but that the physiological costs are paid later at least in mammals (Stier et al. 43). When reproductive investment is artificially increased in birds, it is linked to decreased resistance to an oxidative attack (Alonso-Alvarez et al. 3), lower antioxidant protection (Wiersma et al. 50), or a higher ROM (Christe et al. 2011). However, evidence for a linear cost of reproduction in terms of increased oxidative stress or damage in correlative data from natural populations is more difficult to detect (Nussey et al. 32; Isaksson et al. 23,b; Stier et al. 43; Metcalfe and Monaghan 29; but see also Bize et al. 7).

Finally, the random effect of nest box nested within habitat type had a great influence on all the markers of oxidative stress explaining approximately 7–10% of the variation. This is supported by the very strong positive correlation between social partners’ physiology (of all markers), suggesting that males and females respond equally to environmental influences in their territory, despite a large sex difference in ROM, OXY, and tGSH. Alternatively, great tits mate assortatively with regard to physiology. Whether this is an adaptive response or a consequence of territory quality requires further investigation.

## Conclusion

This study shows that the two commonly used markers of oxidative status in ecological studies – ROM and OXY – are highly correlated in wild great tits, and its variation is explained by similar ecological and individual factors. This is in contrast to the endogenously synthesized antioxidant glutathione, which are not correlated with ROM and OXY, but are still explained by similar factors, but the direction of the effect is in the opposite direction. This finding highlights the need to measure different types of markers to better understand the role of oxidative stress physiology in shaping sex-specific and individual life-history strategies in different environments. Future experimental tests should assess the relative importance of individual versus ecological factors in generating the observed patterns across the breeding season, and thus help to establish the extent to which oxidative stress is important for life-history decisions.
