# Supplementary material for: Opposing effects on glutathione and reactive oxygen metabolites of sex, habitat, and spring date, but no effect of increased breeding density in great tits (Parus major)
Source: Ecol Evol. 2013 Jul 11;3(8):2730–8. doi: 10.1002/ece3.663 (PMC3930037; doi:10.1002/ece3.663)
Supplement: Supplementary file 1 [file ece30003-2730-SD1.docx]

Supplemental material.

**Habitat difference**

Habitat difference in insect abundance (i.e., great tit food) was estimated by even distribution (but randomly placed) of twenty-six water basins (70ˣ45) across the two different habitat qualities (see Hinks 2010 Ph-D thesis). All traps were emptied the same day and number of lepidopterans, arachnids, and dipterans was counted. The study was carried out in 2010 and between the 3^rd^ of May until 7^th^ of June (n = 208, each trap was emptied 8 times). All counted insect groups were significantly more abundant in the deciduous habitat plots compared to the ever-green habitats (n = 208, p = 0.033 – 0.0001). Specifically, *Lepidoptera* caterpillars, which is the most important food source for great tits during the breeding season (Perrins 1991), showed a strong significant difference (n = 208, df_den_ = 27.23, F = 27.23, p < 0.0001, Random effects: trap and tree species explained 10.58% (variance components ± SE, 0.08 ± 0.007) and 1.05 % (0.008 ± 0.04), respectively). Fig. S1 shows the caterpillar abundance and Fig. S2 the seasonal peak in caterpillar abundance in the two different habitats.

**Figure S1.** Small-scale habitat differences in caterpillar abundance. The bars represent mean number of *Lepidoptera* caterpillars caught in traps and the variance with standard errors.

**Figure S2.**  Lepidopteran caterpillar peak in habitats dominated by either ever-green or deciduous tree species. Each peak represents an average from 13 water basin traps. Data are presented as mean with standard error bars.
